# Evaluation of diaphragmatic function in mechanically ventilated children: An ultrasound study

**DOI:** 10.1371/journal.pone.0183560

**Published:** 2017-08-22

**Authors:** En-Pei Lee, Shao-Hsuan Hsia, Hsiu-Feng Hsiao, Min-Chi Chen, Jainn-Jim Lin, Oi-Wa Chan, Chia-Ying Lin, Mei-Chin Yang, Sui-Ling Liao, Shen-Hao Lai

**Affiliations:** 1 Division of Pediatric Critical Care Medicine, Department of Pediatrics, Chang Gung Memorial Hospital at Linkou, Taoyuan, Taiwan; 2 College of Medicine, Chang Gung University, Taoyuan, Taiwan; 3 Department of Respiratory Therapy, Chang Gung Memorial Hospital at Linkou, Taoyuan, Taiwan; 4 Department of Hematology and Oncology, Chang Gung Memorial Hospital, Chiayi, Taiwan; 5 Department of Public Health and Biostatistics Consulting Center, College of Medicine, Chang Gung University, Taoyuan, Taiwan; 6 Division of Pediatric Neurology, Department of Pediatrics, Chang Gung Memorial Hospital at Linkou, Taoyuan, Taiwan; 7 Department of Pediatrics, Chang Gung Memorial Hospital at Keelung, Keelung, Taiwan; 8 Division of Pediatric Pulmonology, Department of Pediatrics, Chang Gung Memorial Hospital at Linkou, Taoyuan, Taiwan; National Yang-Ming University, TAIWAN

## Abstract

**Background:**

The recovery of diaphragmatic function is vital for successful extubation from mechanical ventilation. Recent studies have detected diaphragm atrophy in ventilated adults by using ultrasound, but no similar report has been conducted in children. In the current study, we hypothesized that mechanically ventilated children may also develop diaphragm atrophy and diaphragmatic dysfunction.

**Materials and methods:**

Children who were admitted to the pediatric intensive care unit and were newly intubated for mechanical ventilation were enrolled into this prospective case–control study. Diaphragm ultrasound assessments were performed daily to evaluate diaphragmatic function in the enrolled children until their discharge from the pediatric intensive care unit. Diaphragm thickness and the diaphragmatic thickening fraction (DTF) were measured through these assessments.

**Results:**

A total of 31 patients were enrolled, and overall, 1389 ultrasound assessments were performed. Immediately after intubation, the initial diaphragm thickness and DTF were measured to be 1.94 ± 0.44 mm and 25.85% ± 3.29%, respectively. In the first 24 hours of mechanical ventilation, diaphragm thickness and the DTF decreased substantially and decreased gradually thereafter. After extubation, the DTF was significantly different between the successful and failed extubation groups (P < 0.001), and a DTF value of <17% was associated with extubation failure.

**Conclusions:**

Diaphragm ultrasound is a noninvasive method for measuring diaphragmatic function in mechanically ventilated children. In this study, significant diaphragm atrophy and a decreased DTF were observed within 24 hours of mechanical ventilation. The recovery of diaphragm thickness and the DTF may be a potential predictor of successful extubation from mechanical ventilation.

## Introduction

Critically ill children on ventilation account for one-third of inpatients admitted to pediatric intensive care units (PICUs) [1]. Many recent studies have shown that in adults, mechanical ventilation (MV) may result in atrophy and dysfunction of the diaphragm [2,3]. The reduction of diaphragmatic contraction ability caused by MV is termed ventilator-induced diaphragmatic dysfunction (VIDD), and contributes to a longer weaning time and higher mortality [4]. In healthy individuals, measuring diaphragm thickness during the respiratory cycle can reflect the strength of diaphragmatic contractions [5]. Several recent studies have illustrated that diaphragm ultrasound is a feasible and precise method for evaluating the VIDD [6,7,8]. In adults, the thickness of the right hemidiaphragm and the diaphragmatic thickening fraction (DTF) are used to assess VIDD development [6,7,9]. Furthermore, the DTF is applied as a predictor of successful extubation from MV [10,11].

In the pediatric population, no gold standard has been established for weaning children from ventilators, and no optimal ventilator settings have been identified in PICU practice [12,13]. Conventionally, diaphragm ultrasound is often used to evaluate diaphragmatic palsy in children after cardiac surgical procedures. A recent study on adults revealed that bedside diaphragm ultrasound can accurately estimate the DTF and detect diaphragm atrophy in patients on MV. Moreover, the DTF may be an important predictor of successful extubation [7,10]. However, to date, no study has used diaphragm ultrasound to investigate VIDD development in children. Moreover, the feasibility and accuracy of diaphragm ultrasound in critically ill children on MV remain unknown. In response, this study primarily aimed to detect diaphragmatic contractile dysfunction and atrophy in mechanically ventilated children; we also assessed whether the DTF can be applied to predict successful extubation.

## Materials and methods

### Study design and patients

This prospective case–control study was performed in the PICU of a 3000-bed tertiary hospital (Linkou Chang Gung Memorial Hospital, Taiwan) from February 2016 to January 2017. This study was approved by the Institutional Review Board of Chang Gung Memorial Hospital, and the guardians of all enrolled children signed an informed consent form.

Children aged 1 month to 18 years who were newly intubated for MV were enrolled into this study. Diaphragm ultrasound assessments were performed daily to measure diaphragm thickness and the DTF in the enrolled children until their discharge from the PICU. Patients with underlying neuromuscular disease, chronic respiratory failure, or cerebral palsy, and those who received MV for less than 24 hours, were excluded.

The routine sedative medication of our PICU was midazolam, starting at 2 mcg/kg/min and titrated to obtain a State Behavioral Scale (SBS) of 0/-1. Adjuvant analgesia is provided as 5–25 mcg/kg/min of ketamine. Muscle relaxant medication didn’t administrate in the study. Sedative and analgesia medications were discontinued 12 hours before extubation.

The initial setting of ventilator was pressure control to keep tidal volume with 8–10 mL/kg, SpO2 > 90% and PaCO2 within 35~45 mmHg. We used protective lung ventilation with low tidal volume (4–6 mL/kg) and permissive hypercapnia for patients with ARDS [14].All ventilated children underwent our clinical weaning protocols, which consisted of synchronized intermittent mandatory ventilation (SIMV) plus pressure support (PS) under the following ventilator and clinical conditions: fraction of inspired oxygen (FiO_2_) < 30%, positive end-expiratory pressure ≤6 mmHg, partial pressure of oxygen/FiO2 > 200, respiratory rate ≤20 breaths/min, no vasopressor or sedation drug use, no fever, and stable hemodynamics. Extubation failure was defined as reintubation within 48 hours of extubation due to the following conditions: conscious change, respiratory rate ≥40 breaths/min, blood oxygen saturation <90%, and partial pressure of carbon dioxide >50 mmHg.

### Diaphragm ultrasound

**Fig 1:**
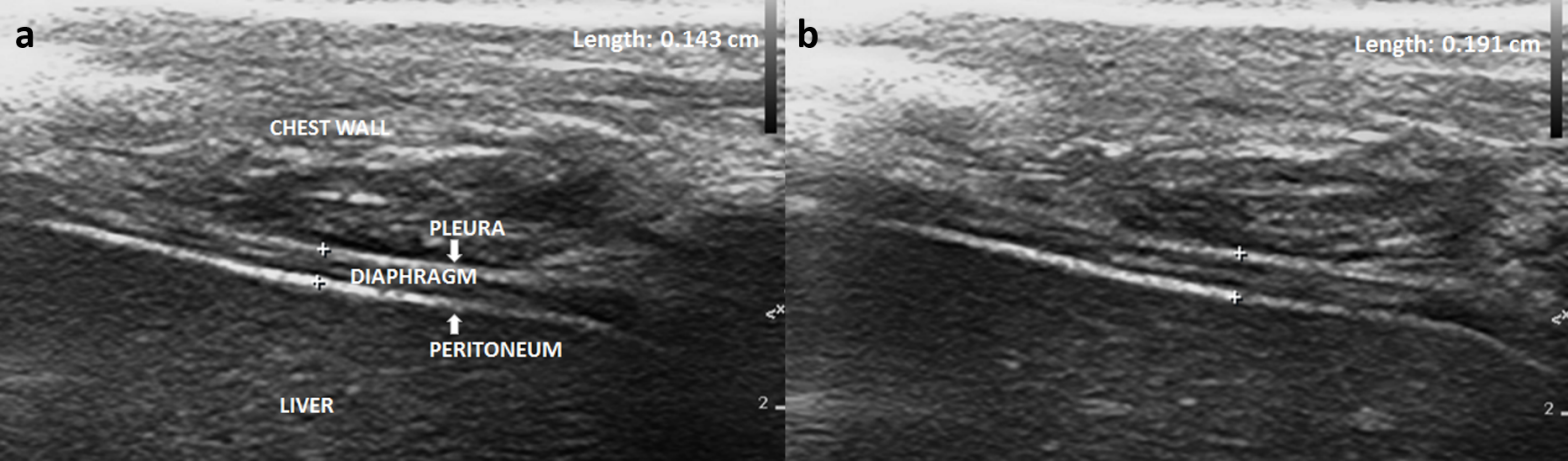
Diaphragm ultrasound at the zone of apposition. (a) Thickness measurement at end-expiration, (b) Thickness measurement at end-inspiration.

Daily diaphragm ultrasound was performed using a PHILIPS (CX50 POC) portable system with a 4–10-HMz linear probe and resolution limit of 0.01 mm. The evaluation of diaphragm thickness was performed as previously described [9–11,15–18]. To obtain a horizontal view of the diaphragm, the linear probe was placed perpendicular to the right chest wall and below the costal margin, in the intercostal space between the eighth and tenth ribs (i.e., between the anterior axillary and midaxillary lines), to observe the zone of apposition of the muscle 0.5–2.5 cm below the costophrenic sinus. The diaphragm was visualized superficial to the liver and consisted of three layers: a nonechogenic central layer and two hyperechogenic layers (the parietal pleura and peritoneum). In the B-mode image, diaphragm thickness was measured from the middle of the pleural line to the middle of the peritoneal line (Fig 1). Subsequently, the index of DTF was defined as

DTF=(End-inspiratorythickness−End-expiratorythickness)/End-expiratorythickness×100%

This index has been proven to be a feasible and accurate parameter for evaluating diaphragmatic functioning and respiratory effort in ventilated and nonventilated patients [6,7,8].

To evaluate the repeatability and reproducibility of diaphragmatic measurements, three measurements were conducted over three daily consecutive respiratory cycles, and the average of the three measurements was calculated. Moreover, two skilled intensivists conducted five measurements on five nonventilated children and five measurements on five ventilated children in the PICU.

### Data collection

In this study, the demographic and clinical data of patients were collected. Daily ventilator settings, including positive end-expiratory pressure, peak inspiratory pressure (PIP), FiO_2_, and respiratory rate, were recorded during the study period.

### Statistical analyses

The demographic data of patients are presented as mean ± standard deviation, median (interquartile range [IQR]), or number (percentage). The intraobserver repeatability and interobserver reproducibility were assessed using the intraclass correlation coefficient. Comparisons between the two groups were conducted using the Wilcoxon rank-sum test. The change in the DTF over time was determined using the generalized estimating equation [19], which accounts for possible correlations in repeated measurements within a patient. All statistical analyses were performed using SPSS (version 22.0; SPSS Inc., Chicago, IL, USA), and a P value of <0.05 was considered statistically significant.

## Results

### Patient characteristics

Thirty-one mechanically ventilated children were enrolled in this study. The median MV duration was 7 days (IQR, 4–15), which is slightly higher than the 6 days reported by Khemani et al [20]. The median PICU stay was 9 days (IQR, 7–23) and the total PICU stay of all 31 patients was 463 patient-days, during which 1389 ultrasound assessments were performed. The median age of the enrolled children was 3 years, and 54.8% were boys. Patient characteristics are summarized in Table 1. For all patients, the ventilator mode was initially set as pressure control.

**Table 1:** Demographics of intubated children admitted to the PICU.

**Baseline data**	
No. of patients	31
Age (yr)	3 (0.5–11.9)
Male	17 (54.8)
Weight (kg)	22.5 ± 20.8
**Admission diagnosis category, n(%)**	
Pulmonary	12 (38.7)
Sepsis	6 (19.4)
Cardiovascular	2 (6.4)
Neurologic	9 (29.1)
Other	2 (6.4)
**Initial Ventilatory setting**	
Pressure control ventilation	31
Set respiratory rate (min-1)	25.6 ± 8.9
PIP (cmH2O)	21.4 ± 5.1
PEEP (cmH2O)	6.4 ± 1.9
FiO2 (%)	53.9 ± 26.1
**Outcomes**	
Days of mechanical ventilation	7 (4–15)
ICU length of stay	9 (7–23)
ICU mortality (%)	7 (22.6)

Result are presented as median (IQR), mean±SD, or number (percent)

PIP = peak inspiratory pressure; PEEP = positive end-expiratory pressure; FiO_2_ = fraction of inspired oxygen; PICU = pediatric intensive care unit

### Ultrasound measurements

Table 2 presents the average of daily ultrasound measurements conducted during the 7 days of MV. The data of all ultrasound assessments were pooled for the intubation and extubation groups, which the intubation group contained 353 patients-days and the extubation group contained 110 patients-days. Thus, the end-inspiratory thickness of the diaphragm and the DTF were significantly higher in the extubation group than in the intubation group (1.86 ± 0.46 vs 1.66 ± 0.44 mm and 24.9 ± 3.3 vs 14.8% ± 4%, respectively; both P < 0.001).

**Table 2:** Average of daily ultrasound measurements over time in the first week of MV.

	Day1 (n = 31)	Day2 (n = 28)	Day3 (n = 25)	Day4 (n = 23)	Day5 (n = 20)	Day6 (n = 17)	Day7 (n = 15)
End-inspiratory thickness (mm)	1.92±0.48	1.75±0.41	1.74±0.44	1.67±0.46	1.66±0.42	1.69±0.39	1.69±0.45
End-expiratory thickness (mm)	1.52±0.38	1.5±0.35	1.52±0.39	1.46±0.4	1.45±0.37	1.49±0.36	1.49±0.42
Diaphragmatic thickening fraction (%)	25.8±3.3	16.4±4.2	14.5±2.6	14.3±2.9	14.3±2.6	14.2±2.8	13.5±3.7

The clinical characteristics of children with successful and failed extubation are shown in Table 3. In this study, six children did not receive extubation due to death and the DTF after extubation was significantly higher in the successful group (P < 0.001).

**Table 3:** Characteristics of successful and failed extubation groups.

Baseline data	Success	Failure	P value
No. of patients	22	3	
Age (yr)	1.8 (0.5–5)	2 (1–11)	0.913
Male	10	1	
Weight (kg)	16.5 ± 15.7	17.3 ± 20.5	0.938
**Admission diagnosis category, n(%)**			
Pulmonary	7 (31.8)	3 (100)	0.024
Sepsis	4 (18.2)	0	0.42
Cardiovascular	1 (4.5)	0	0.71
Neurologic	8 (36.4)	0	0.21
Other	2 (9.1)	0	0.58
**Initial Ventilatory setting**			
Set respiratory rate (min^-1^)	24.8 ± 8.1	32.7 ± 11	0.256
PIP (cmH2O)	20.1 ± 4.8	23.7 ± 4.5	0.193
PEEP (cmH2O)	5.9 ± 1.5	6±1	0.214
FiO2 (%)	45 ± 20.5	73.3 ± 11.6	0.032
**Diaphragmatic measurements**			
Initial mean thickness of diaphragm (mm)	1.89 ± 0.36	1.78 ± 0.29	0.738
DTF at Day 1 (%)	26.3 ± 3.5	23.1 ± 1.9	0.094
DTF before extubation (%)	15.6 ± 2.7	14.2 ± 4.02	0.451
DTF after extubation (%)	23.9 ± 3.2	14.5 ± 1.9	<0.001
**Outcomes**			
Days of mechanical ventilation	10.1 ± 9.4	24 ± 18.7	0.065
ICU length of stay	14.8 ± 11.7	25 ± 20.4	0.193
ICU mortality, n(%)	1 (4.5)	0	0.712

Result are presented as median (IQR), mean±SD, or number (percent)

PIP = peak inspiratory pressure; PEEP = positive end-expiratory pressure; FiO_2_ = fraction of inspired oxygen; PICU = pediatric intensive care unit; DTF = diaphragmatic thickening fraction

**Fig 2:**
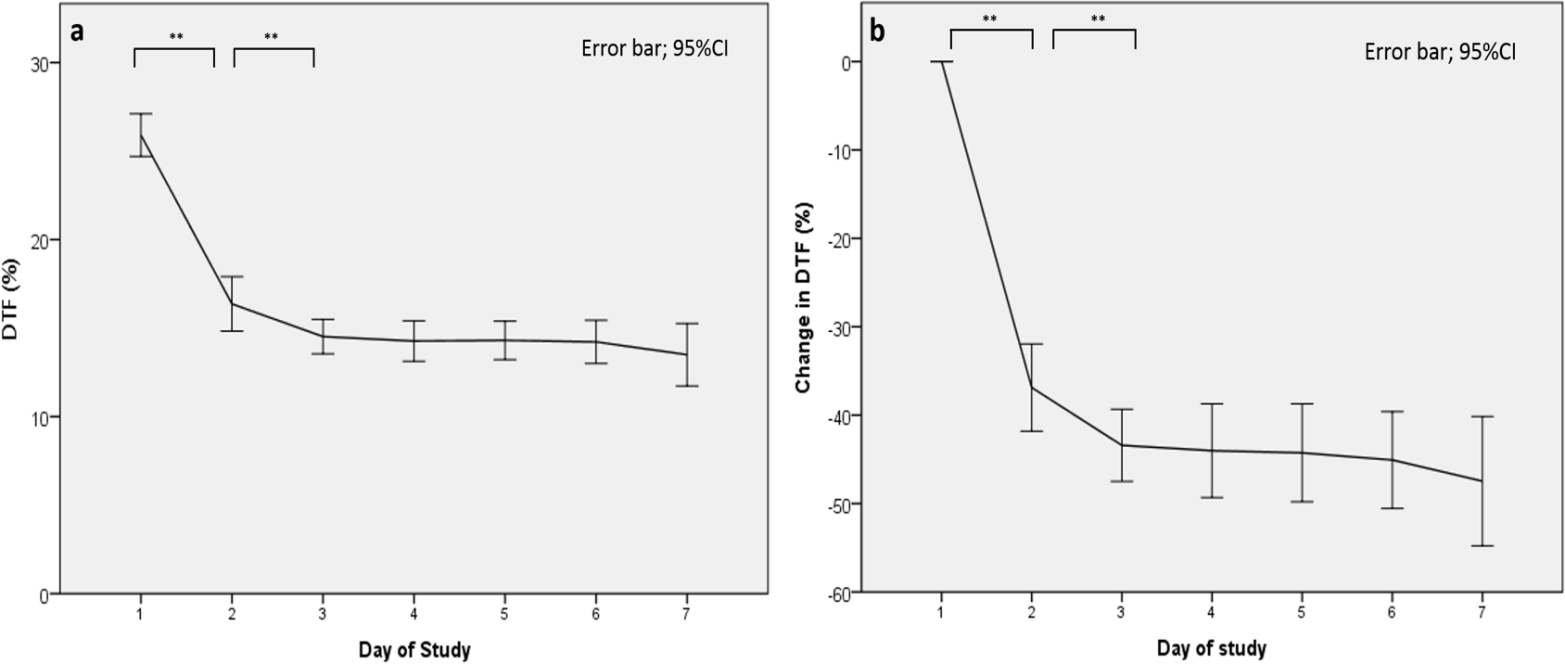
Average of daily ultrasound measurements over time in the first week of MV. **(a) Mean daily diaphragmatic thickening fraction (DTF), (b) change in DTF**. CI = confidence interval; **P < 0.005.

The DTF decreased substantially during the first 2 days of MV and gradually decreased thereafter (Fig 2A and 2B). After intubation, most patients exhibited an initial mean decrease of 9.4% for the DTF, from 25.8% on day 1 to 16.4% on day 2 of intubation. Subsequently, the DTF decreased from 16.4% on day 2 to 13.5% on day 7, with an average decrease of 0.58% per day (Fig 2A). As shown in Table 2, the mean diaphragm thickness also decreased from 1.92 mm on day 1 to 1.75 mm on day 2 of intubation (a 8.8% decrease). Subsequently, diaphragm thickness measured from day 2 to day 7 of intubation indicated a gradual reduction of the thickness, with an average decrease of 0.68% per day.

**Fig 3:**
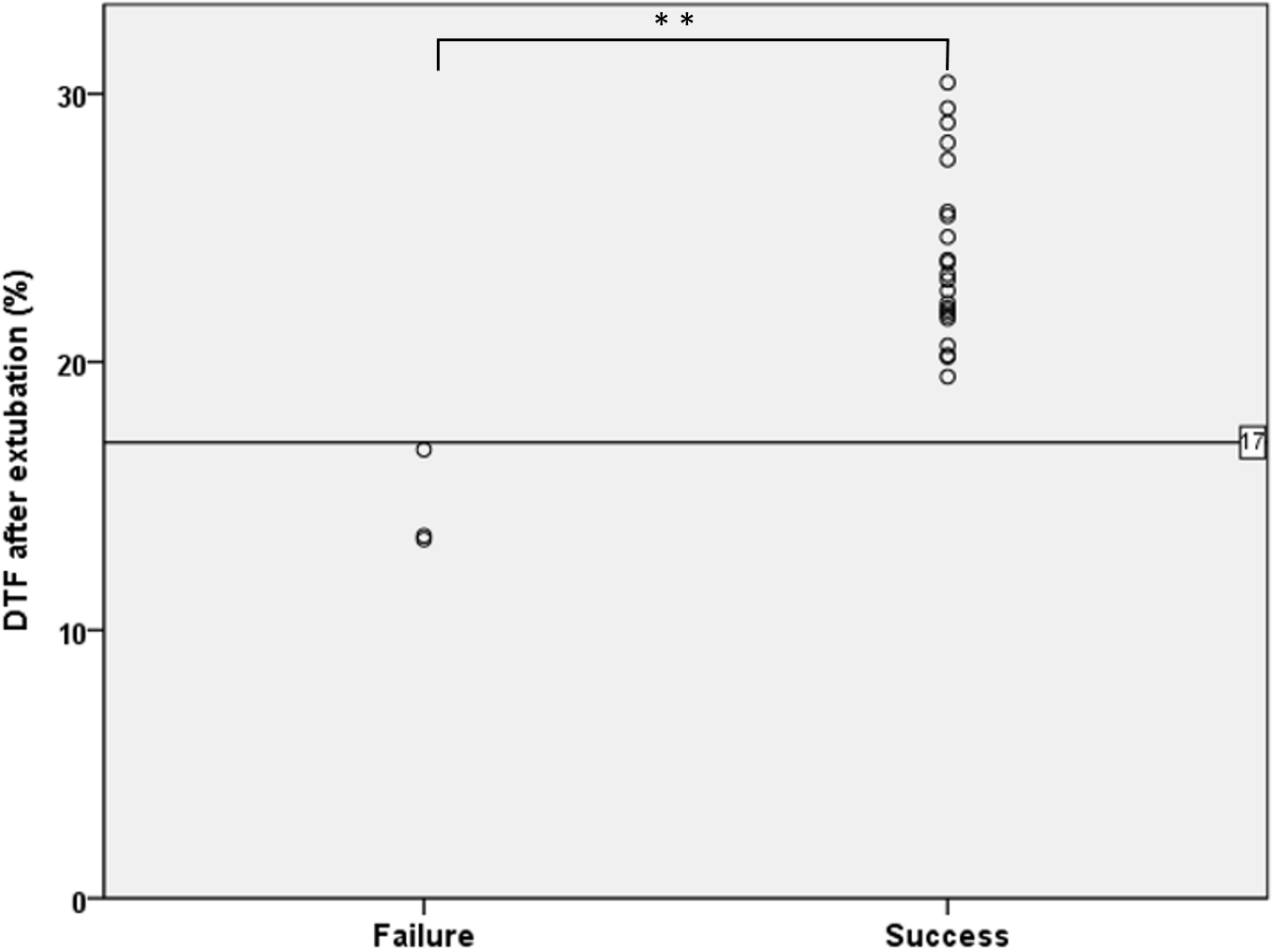
Diaphragmatic thickening fraction (DTF) after extubation in children with failed and successful extubation. **P < 0.005.

A significant difference was observed in the DTF between the successful and failed extubation groups. Moreover, all the three DTF were less than 17% (16.7%、 13.3%、 13.5%) in the failed group (Fig 3). All of the 3 failed cases met the Berlin Definition of severe ARDS. The first failed case was a 2-year-old child, admitted due to pneumonia with *Pseudomonas aeruginosa* infection. The worst oxygenation ratio (PaO2/FIO2) was 63. Sedative medication was administrated for 43 days and the total duration of intubation was 44 days. The second case was a 1-year-old child with underlying tracheomalacia and hyperteactive airway, admitted due to suspected viral pneumonia. The worst oxygenation ratio was 88. The third was a 11-year-old with the underlying of T-cell lymphoma admitted due to severe septic shock and bilateral pneumonia. The worst oxygenation ratio was 70. Sedative medication was administrated for 13 days and the total duration of intubation was 53 days.

### Repeatability and reproducibility

The correlation coefficients of interobserver reproducibility of the DTF, end-inspiratory thickness, and end-expiratory thickness were 0.9 (0.649–0.947), 0.996 (0.983–0.999), and 0.996 (0.985–0.999) respectively. Similarly, the correlation coefficients of intraobserver repeatability of the DTF, end-inspiratory thickness, and end-expiratory thickness were 0.828 (0.803–0.851), 0.915 (0.902–0.927), and 0.913 (0.899–0.925) respectively. All correlation coefficients were above 0.75, which indicated good agreement [21].

## Discussion

This study is the first to analyze the role of diaphragm ultrasound in the weaning policy of children on MV, and two critical findings were observed. First, atrophy of the diaphragm and reduction of the DTF were immediately observed within the first 24 hours of MV initiation. This finding is consistent with that of previous studies in adults [2,3,22]. Second, the recovery of the DTF after extubation may be an initial predictor of successful extubation from MV potentially.

Recent studies have extensively demonstrated disuse atrophy and contractile dysfunction of the diaphragm in mechanically ventilated adults [3,22,23]. The biopsy reported that MV adversely affect the diaphragmatic fiber architecture (decreased slow- and fast-twitch fibers, atrophied diaphragmatic fibers, and disrupted sarcomere structure) histologically, and the mitochondrial respiration also been damaged biochemically [3,22]. Reactive oxygen species overproduction induced by impaired mitochondrial respiration may also induce oxidative damage in diaphragmatic proteins and lipids. Moreover, altered diaphragmatic gene expression secondary to MV results in the dysregulation of diaphragmatic protein synthesis and the activation of proteolysis, which accelerate protein breakdown and fiber atrophy [22,23]. Finally, the diaphragmatic force produced decreases, contributing to diaphragmatic contractile dysfunction.

VIDD detection has been extensively described in adults; however, no clinical parameter for VIDD detection has been established in ventilated children. The current study demonstrated the DTF in children averaged 25.8% immediately after intubation, consistent with the DTF range of 25%–40% during resting tidal breathing in healthy adults [6,24]. Moreover, the mean DTF decreased substantially within the first 2 days of intubation and then decreased steadily from day 3 to day 7. The mean diaphragm thickness also decreased significantly within the first 2 days and decreased steadily thereafter. This initial decrease is more rapid than the average decrease of 6% per day in ventilated adults reported by Grosu et al [25]. The more rapid decrease in the DTF and diaphragm thickness in mechanically ventilated children may be attributed to the presence of fewer type 1 fibers (slow-twitch, high-oxidative), which have higher oxidative capacity [26]. The loss of sparse type 1 fibers immediately after intubation may result in poor resistance to diaphragmatic fatigue in children and the initial substantial deterioration of diaphragmatic contractions.

For all patients, the mean DTF before extubation was approximately 16%, but no significant difference was observed in the DTF between the successful and failed extubation groups in our study. This finding contradicts that of Ferrari et al, in which DTF > 36% during the forced inspiration breath was a favorable predictor of successful extubation in adults [10]. Indeed, our study found that the DTF did not recover to the baseline value before extubation, which may be due to the latency of diaphragmatic recovery under the weaning mode of SIMV + PS [7]. Although a spontaneous breathing trial under a T-piece is the routine weaning policy in adults, it is not recommended in children due to the high resistance of ventilator circuits secondary to the small diameters of the endotracheal tube [13]. This study found that DTF < 17% immediately after extubation could predict the need for reintubation potentially. Although low validity was obtained for the DTF cutoff value for predicting extubation failure (only three cases of extubation failures were noted), this cutoff can provide useful clinical information for predicting the need for reintubation. Furthermore, the postextubation DTF may be a favorable indicator for further interventions, such as noninvasive positive pressure ventilation, to improve the diaphragmatic contraction force after extubation and to avoid reintubation [7].

The three cases of extubation failure in our study had higher initial ventilator settings, including a higher FiO_2_, higher respiratory rate setting, higher PIP, lower DTF, and lower diaphragm thickness. Both predisposing dysfunction of the respiratory system and the prolonged course of dyspnea in these three patients may have exacerbated diaphragmatic muscle wasting before intubation. Furthermore, other risk factors for extubation failure, including longer intubation duration (>15 days) and younger age (<2 years), were observed in two patients; underlying leukemia, increased sedation (>10 days), and inotropic agent use were also noted in one patient. All of these risk factors have also been reported by Kurachek et al [27] and Fontela et al [28].

Consecutive real-time diaphragmatic ultrasound can easily be performed in intubated children. In the current study, it was simple and easy for clinicians to practice and monitor diaphragmatic functioning by using the B-mode of diaphragmatic ultrasound. The high repeatability and reproducibility are comparable with those in previous studies in adults [6,7]. In addition, daily ultrasound measurement could be performed as rapidly as within approximately 5–10 minutes. Thus, diaphragm ultrasound is a feasible tool to monitor diaphragmatic activity and atrophy in mechanically ventilated children.

Our study has some limitations to note. First, we studied a relatively small population, although the case number is comparable with that of previous studies with similar findings [9,29]. Second, in this study, we did not analyze other variables that might affect diaphragmatic contraction, such as severity scores of disease, inotropic agent use, sedatives or neuromuscular blocking agents, and various disease etiologies. However, none of these aforementioned variables were determined to exert statistically significant effects on diaphragmatic function in a previous study [7]. Third, the postextubation DTF can’t be a powerful predictor for successful extubation due to small case numbers. However, it can be a potential predictor for noninvasive or invasive positive pressure ventilation. Finally, because no reference values of diaphragm thickness and the DTF have been established in children, it is difficult to determine whether the initial diaphragmatic function of our enrolled children is abnormal or not. Based on our preliminary study in normal children, the postextubation diaphragm thickness and DTF of children with successful extubation were comparable with those of the normal population. However, studies with larger sample size should be conducted to establish the reference values and determine the difference in diaphragmatic function between children with different ventilation support.

In conclusion, diaphragm ultrasound is a promising tool for assessing diaphragmatic function in mechanically ventilated children. The end-inspiratory diaphragm thickness and DTF measured using ultrasound can provide useful information for evaluating diaphragmatic function and its contribution to respiratory work. Furthermore, these measured parameters may be used to titrate optimal ventilator settings. In the children in this study, diffuse diaphragm atrophy and decreased DTF were immediately observed within the first 24 hours of MV. The recovery of diaphragm thickness and the postextubation DTF may be useful for predicting successful extubation from MV potentially.
